# On Cancer of the Tongue

**Published:** 1896-08-08

**Authors:** J. Lacy Firth

**Affiliations:** Assistant Surgeon to the Bristol General Hospital


### Medical Progress and Hospital Clinics.

[*The Editor will be glad to receive offers of co-operation and contributions from members of the profession. All letters should be addressed to* The Editor, at the Office, 428, Strand, London, W.C.]

### ON CANCER OF THE TONGUE.

By J. Lacy Firth, M.S., F.R.C.S., Assistant Surgeon to the Bristol General Hospital.

Mr. Watson Cheyne, in his recent lectures on the "Objects and Limits of Operations for Cancer" (*Lancet*, vol. I., 96) has very forcibly drawn attention to the greatly improved results which are to be expected from these operations in the future when performed in the thorough manner dictated by modern anatomical and pathological research. Three years ago, Mr. Cheyne had operated on twenty-one cases of scirrhus of the mamma in the modern manner, and twelve of these cases, or 57 per cent., have not as yet had any recurrence, and may be regarded as radically cured; and he points out that the older surgeons, operating by older and less thorough methods, could not show as many cures, though the number of their cases exceeded a hundred. These splendid results have been achieved by removing the affected organ and the axillary lymphatic glands *en masse*, together with a wide area around them of healthy tissue and the whole length of any muscular fibres implicated.

In the treatment of epithelioma of the tongue, Mr. Cheyne confidently predicts improvement in the results when similar methods of operation are adopted. The sublingual and submaxillary and lymphatic glands should always be removed on the affected side, and if the muscles of the tongue are infiltrated they must be removed down to the hyoid bone, following Heidenhain's law. Success in all this class of operations can only be secured by getting well beyond the diseased area. The watchword should be "thoroughness." The more localised the diseased area the more possible it is to get well beyond it. In all cancer cases, if left alone, a time comes when it is impossible completely to eradicate the disease, and the more rapid the growth—that is, the more malignant it is—the sooner is this hopeless period reached. The best results in breast cancer are obtained when the growth is of the atrophic form, because in this form there is the least tendency to dissemination.

Cancer of the tongue is, unfortunately, an intensely malignant growth, spreading rapidly, and involving the lymphatics early. On the other hand, metastatic diffusion is uncommon. The rapidity of its growth is favoured by the heat and moisture of the mouth, the constant movement of the organ affected, and its frequent irritation during the mastication of food. Hence it is of primary importance that operations for the removal of this disease should be performed early in its evolution, and the methods of diagnosis of its initial stages demand the most careful study. Writing in 1872 (*British Medical Journal*, vol. 1, p. 5), Mr. Hutchinson says: "I have often explained and enforced the doctrine of a pre-cancerous stage of cancer. According to this doctrine, in most cases of cancer of the penis, lip, tongue, and skin, &c., there is a stage—often a long one—during which a condition of chronic inflammation only is present, and upon this the cancerous process becomes engrafted." Obviously this is the stage during which operations should be performed to yield the best results. Unfortunately, there is great difficulty often in differentiating those states of chronic inflammation which are pre-cancerous and certain to produce cancer and those which are not certain to do so. If the pre-cancerous state could be recognised with certainty, and if radical treatment for it were at once adopted, well-developed cancers of the tongue, with all the misery they bring in their train, would soon cease to afflict humanity. That is a Utopian dream; but the more surgeons and patients bestow attention upon the pre-cancerous stage of cancer and its diagnosis, the smaller will become the number of victims who fall under the baneful attacks of the disease. Picture, on the one hand, the miseries of these victims, and, on the other, the slight inconveniences entailed as a result of early operation. As Jacobson says ("Operations of Surgery," p. 361), "this condition of the "tongue renders the patient a nuisance to himself and others with the disgusting fœtor, the constant dribbling of foul saliva which cannot be swallowed, the incessant weary, racking, aching of tongue, ear, face, and teeth, day and night, lit up into agonising flashes when the parts are touched or moved." The same writer (Guy's Hospital Reports, 1889) gives notes on the after condition of a case of excision of half the tongue, which show that speech remained perfectly intelligible though slightly imperfect, and the only other defects were inability to protrude the tongue beyond the teeth and slight impairment in mastication. The advantages conferred by early operation are at the present day being enjoyed by many individuals who have been permanently cured; but cancer of tongue remains a lamentably frequent disease, and the majority of cases when brought under the notice of operating surgeons are in so advanced a state that there is but faint hope of the possibility of extirpating the whole diseased area. It is sad to reflect that in every such case the period in the evolution of the disease when radical cure might have been effected has been allowed to slip by.

To be most fruitful in practical result differential diagnosis should be possible of the following pathological conditions of the tongue:—

(*a*) Conditions which will not cause cancer.

(*b*) Pre-cancerous conditions of two classes, viz.: (i.) Conditions which may cause cancer; and (ii.) Conditions which are actually producing cancer or certainly will.

(*c*) The initial forms of actual cancer.

We will briefly consider these varieties of disease, taking them in inverse order. The initial forms of actual epithelioma of the tongue are the same as those of epithelioma in other parts, *e.g.*, the cervix uteri. They are three, and the following is Mr. Barker's description of them ("Holmes System of Surgery," vol. ii., p. 592)—(1) The small, hard, sharply defined pimple, or knot, a little raised, perhaps, but smooth on the surface, first observed just beneath the coverings, but in the substance of an otherwise healthy tongue, usually at one side or actually on its border; (2) the small abrasion or crack, more likely to be on the upper surface of the organ, starting without any previous induration, but very often from a blister; (3) the prominent or papillomatous form, looking benign at first, but developing into typical epithelioma later, situated, as a rule, on the dorsum of the tongue somewhat to one side and far back.

Mr. Barker points out that it is the small deep deposits which lead the soonest to lymphatic infiltration.

The varieties of the pre-cancerous conditions as enumerated by Mr. Jacobson are as follows: (1) Old persistent glossitis with hypertrophy and sulci. The cancer often begins as a rawness at the bottom of one of the sulci. (2) Leucoplakia with (a) persistent rawness of a patch; (b) formation of a knot, or lump, of induration in one of them. (3) Icthyosis. This is a variety of leucoplakia in which the patches are warty with a projecting and irregular surface. (4) Bald tongue (red glazed tongue?). Here the normal papillary surface of mucous membrane is replaced by what is approximately scar tissue. (5) Warts. A very suspicious form of wart formed by low, small papillæ grouped on a slightly indurated disc, is described by Hutchinson (*Clinical Journal*, Vol. I.). (6 and 7) Fissures and cracks. (8) Ulcers. The ulcers may be of syphilitic origin, or originate from the irritation of sharp teeth, edges of dental plates, or deposits of tartar. To the above may be added (9), any scar in the tongue.

The conditions of the tongue which do not assist in producing cancer are its more acute affections. These will not cause cancer unless they assume one of the pre-cancerous forms of disease as above enumerated.

Those diseased states of the tongue which possibly cause cancer, and those which certainly will, do not exhibit such positive differences in physical characters as to enable surgeons to effect a differential diagnosis between them. This is the main reason why epithelioma of the tongue is so often allowed to progress to full development. This uncertainty of diagnosis only too often induces the practitioner to temporise. The patient with his hopes supported by the uncertainty, and with a dread of what he conceives to be a horrible mutilation, readily acquiesces in the delay, and thus that valuable period of time during which an operation might effect a radical cure is allowed to pass, and muscular and glandular cancerous infiltration occurs. There are but few cases on record in which a cure has resulted when the operation has been undertaken after glandular enlargement was present, but many operations undertaken for the pre-cancerous condition, or for cancers of early development, have cured their victims.

The causes of the fatal delay so often seen is the dread of operation by the patient, and the diagnostic uncertainties that beset the medical adviser. These will be best combated when the former is dispelled by reassurances based upon the recent improvements in the results of the operations as shown by statistics, and the latter by the cultivation of a readiness to take alarm at the earliest signs of malignancy.

These earliest signs are suspicious induration, persistent excoriation or ulceration, however small, and the characters of the cells removed by scraping the surface of the suspected area. This last valuable method we owe to Mr. Butlin. He showed (sarcoma and carcinoma, p. 154) that the cells obtained by lightly scraping the surface of the suspicious patch with the back of a scalpel when examined microscopically are in those cases undergoing cancerous change more variable in size and shape, the great majority being tailed or oval and withered up, and have larger nuclei than the normal epithelial cells, or than the cells from a simple inflammatory ulcer. Also cell nests, or fragments of cell nests, may be observed.

